# The Status of Our Profession and Its Educational Methods in England, as Shown in Recent Publications

**Published:** 1900-01

**Authors:** William H. Trueman

**Affiliations:** Philadelphia


### THE STATUS OF OUR PROFESSION AND ITS EDUCATIONAL METHODS IN ENGLAND, AS SHOWN IN RECENT PUBLICATIONS.[1]

BY WILLIAM H. TRUEMAN, D D.S., PHILADELPHIA.

In fulfilling an obligation assumed when accepting honorary membership in your body, and an assignment to your Committee on Dental Literature, I have elected to present "The Status of Our Profession and its Educational Methods in England, as shown by Recent Publications." The matter presented has been gathered mainly from the English dental journals for 1898, in the effort to form an *exposé* of the general trend of thought there expressed upon matters of common interest effecting the status of our profession, its

[1] Read before The New York Institute of Stomatology, October 3, 1899.

educational interests, its scientific standing, and its general tone. It has seemed to me that this would be in line with your committee's work, and probably would not, as these journals are not widely read in this country, trench upon work already done or in contemplation, and that it might be of interest to have in concrete a general idea, even limited as a paper like this must necessarily be, of what our brethren on the other side have been doing in matters upon which we are all so keenly alive.

We will first turn to the *Journal of the British Dental Association*, for to this journal, published by the Association, and its official organ, by virtue of this, in all matters concerning strictly professional affairs, must be accorded first place among English dental periodicals. In addition and apart from this, however, it has always been well edited, conservative yet progressive, thoroughly professional in tone, and well merits this position as an official exponent of English dental thought. It has, I am sorry to say, no counterpart in the United States. That this is so seems an oversight on the part of our national associations, past and present. For a few years the American Association of Dental Surgeons published the first dental journal of which we have any record. It is much to be regretted that they ever allowed it to pass out of their hands. It is more than an oversight, it is a blunder, that its successors have so far failed to remedy this mistake by providing for themselves an official organ and for the profession a high-toned, independent dental journal, as the British Dental Association has done, instead of year after year making merchandise of their proceedings. For a number of years the *Journal of the British Dental Association* was published at a financial loss. Since 1895 it has proved an increasing source of revenue. Far more important than the mere money consideration, however, it has been a means of keeping the Association in touch with its constituents, and has provided for the dental profession in Great Britain a journal entirely free from commercial interests and wholly within its control.

An editorial in the January number, entitled "The Educational Business of the General Medical Council," is instructive. It complains very mildly of the Medical Council's tardy action upon desired changes in the dental curriculum, and explains that the method adopted by the council necessarily entailed a certain amount of delay. First, a visiting committee was appointed to inspect and report upon the work of the various examining bodies. These reports, after being formally received, were submitted to the examining bodies concerned for any criticism or suggestions that they might have to make. When these are returned, they are referred for discussion to the next session of the General Council. This is the usual course with such matters, and it may be that several sessions pass before final action is taken. This, as the Council meets but twice a year, takes considerable time, but insures thorough and careful work. Any supposed dereliction of duty in the schools or examining bodies is not there, as it is with us, at once anonymously ventilated in the dental journals or societies by irresponsible parties, in such a manner that, while a slur is cast upon all alike, neither the schools nor the examining boards implicated, nor those to whom the information may be of value, are at all benefited or enlightened. The conservative, yet effective, procedure of the British General Medical Council is far more dignified: fair, open, and just to all concerned. When the matter is finally reached, after at least six months' careful thought, the conclusions are far more satisfactory to the teachers, more helpful to the taught, and more useful to those who are desirous that their pupils shall receive the best possible professional training.

They have on the other side the same knotty problems that confront us here. There is a very general desire to elevate and make more of the dental profession, but all are not agreed as to the ways and means. The educational problem has been for some years the subject of anxious thought. There is a marked diversity of opinion regarding the changes desirable and the changes possible,—the standard the *powers that be* are willing to sanction, that which leaders of the profession demand, and that which the schools and the examining bodies deem expedient for their respective bailiwicks. There has not been, and for some time cannot be, that uniformity which should exist, in view of the fact that a license to practise, or rather to assume the title of dentist, granted by either one of the several licensing bodies is equally good in any part of the realm. On these points the medical and the dental professions are equally agitated. The dental profession is not in Great Britain free and independent and in a position to control its own affairs, as it is with us. They are legally so blended—the two professions—that the dental is the ward of the medical, and until a little more than a year ago had no voice in the council that regulates even to the minutest detail its legal and educational affairs, except through the courtesy of its medical friends. This will explain why the remarks that follow are taken from addresses by medical men, writing upon medical topics. They were, while speaking in their own interests, equally advancing the interests of their voiceless and dependent ward. In this controversy one party aggressively demands a "single portal" for each profession,—that is, that for medicine and for dentistry the course of instruction in all the schools teaching these sciences and the standard of all the examining bodies granting medical and dental qualifications shall be exactly the same; just as though there was for the medical profession but one school and but one examining board, and a corresponding school and examining board for the dental profession. To accomplish this is not simply a question of levelling up or of levelling down. It would require some schools of excellent repute to entirely recast their course of instruction, and would thereby introduce much confusion, not only in the schools themselves, but also in their relation to other schools and to other professions, without the slightest advantage or any real educational advance. It would also require the examining bodies from which their students receive their degrees to radically change their methods without any material upward or downward change in their standards. These naturally object to any immediate radical change, but suggest that it may be brought about in course of time.

An address by Mr. Victor Horsley, F.R.C.S. (Eng.), F.R.S., entitled, "The Medical Acts of Parliament: As they are and as they ought to be,"[1] is an excellent presentation of the subject from the stand-point of the aggressive party. Mr. Horsley is a member of the Medical Council who has always shown a friendly and active interest in the dental profession. While his remarks in this address are upon the Medical Act, as I have before stated, that and the Dental Act are so closely related that his address would be just as appropriate if, wherever it occurs, the word dental were inserted in place of medical. He first treats at some length of a peculiarity of both the Medical and Dental Acts. Neither prohibits or punishes, except by implication, practice by unqualified persons. In this they have been a disappointment. It was presumed that a law which required that all who assumed to practise either art should, after complying with certain legal requirements, be registered as qualified practitioners would do more than protect the title designating the calling of those who obeyed it, and do more than forbid the use of the title and due process of law in collecting fees by those who disregarded it. It was presumed, indeed, that it was especially aimed at the *practice* of

[1] Journal of the British Dental Association, January, 1898, page 45.

either profession by those not properly qualified. Practically, so far as it has been expounded by judicial decisions, it permits any ignoramus to practise, provided he be careful not to use the title. In the case of British Dental Association *vs.* Herbert Manning, heard at Lough, Lincolnshire, September 3, 1898,[1] Mr. R. W. Turner, in stating for the prosecution the law involved in the case, said, "A person shall not be allowed to take the title or use the title of dentist, either alone or in combination with any other word or words, or of dental practitioner, or any name, title, or description implying that he is registered under this Act, or that he is a person specially qualified to practise dentistry, unless he is registered under this Act. An amendment declares that the words 'title or description,' where used in the Dental Act, include any addition to a name or designation or description, whether expressed in words or by letters, or partly in one way and partly in another." In this case, as in others, the conviction secured was for using the title, *not* for engaging in practice. Now, Mr. Horsley contends that this is all wrong; that a law which requires a man to spend years in acquiring knowledge and skill to become under it a qualified practitioner should protect him in the practice of his art from the competition of those who have not so done. He thinks that the law as it stands will bear this construction. He admits, however, that the General Medical Council and magistrates do not take that view of it, and that the legal advisers of the Medical Defence Union have suggested that it would be expedient to get an amendment to the Act, so as to make it clear that a person who was not registered was not entitled to practise, and that if he did so he would be punished. He argues at some length in support of his contention, and chides the Medical Council for being guided in these matters by their legal advisers. He urges that they should put their own construction upon the law it is their duty to enforce, and, selecting a case where this issue is involved, prosecute it to a finality. Calling attention to the so-called "overcrowding" in the profession, he argues that this could in part be prevented by raising the standard of the preliminary examinations, and contends that these—indeed, all the examinations, from preliminary to final—should be absolutely uniform throughout the United Kingdom, and conducted by the General Medical Council. He suggests, further, that they should confer upon the successful

---

[1] Journal of the British Dental Association, vol. xix., October, 1898, page 739.

candidate the state qualification to be registered, carrying with it the title of "doctor." This is known as the one-portal system, and is opposed in the Medical Council by the representatives of the corporations or guilds, for reasons financial and otherwise.

In an address upon "Legislation as a Remedy for Medical Grievances," Mr. R. Brudenell Carter, F.R.C.S. (page 109, February number of the same journal), gives the other side of these controverted questions. It is, indeed, an answer to Mr. Horsley's remarks. Mr. Carter tells us that after a long experience as a general medical practitioner he, as surgeon to the eye department of St. George's Hospital, and later as a member of the editorial staff of two medical journals, has been brought into intimate and friendly relations with general practitioners in all parts of the country. Since when, in the early fifties, medical legislation was first broached, he has had ample opportunity to become well acquainted with all the varied phases of medico-political questions. Since 1887 he has been a member of the General Medical Council. An address coming from a man with so wide an experience, so practical in his ideas, so excellent and pertinent throughout, merits a careful and thoughtful reading. I have room here for only a few of the more prominent suggestions, and have endeavored to select those which are of more than local interest. He says, "The members of every calling or profession find something in their relations with the public or with their more especial *clientèle* which might be amended, and it will also be conceded that, as the wearer of the shoe knows best where it pinches, so the members of every profession know their own grievances, and think more of the evils these grievances produce than they would of the grievances of other people.

"In these circumstances I am disposed to look on the consciousness of grievances on the part of the members of any profession somewhat as a sign of healthy vitality, and I should feel doubt as to the future of a society in which grievances and a determination to remedy them did not exist. But the present grievances of the medical profession, in my humble judgment, much transcends what we may describe as normal, and are becoming year by year more oppressive.

"If they do not arise from, they are least intensified by, the fact that members of the profession of late years have been increasing in numbers with a rapidity previously unknown, whilst at the same time the demand for their services has in many directions been diminishing. The general result of increased knowledge has been greatly to diminish the amount of illness occurring, and in surgical cases, particularly, to curtail the duration of illness.

"Just to take an example: A woman with an ovarian tumor would have been likely to sink into the grave after some four or five years of lingering illness, during which she would have been constantly dependent for the relief of suffering on the general medical practitioner. At the present time she undergoes an operation, and in a month is restored to health.[1] I have seen it stated that there are fourteen thousand general practitioners in England and Wales. If this be true, there must be more than eight thousand physicians and surgeons, in a strict sense, specialists. Can we wonder that men of all classes find it difficult to keep the wolf from the door, or that a few stoop to practices which they can only excuse by saying 'my poverty, but not my will, consents'? It must not be supposed that I have the slightest sympathy with Mr. Horsley's desire to check entrance into the profession. This would be a mere trades-union device; medical students are in large proportion the sons of medical practitioners, and while due provision should be made for raising the standards in a degree commensurate with the progress of the science, parents are entitled to bring their sons into the profession on about the same terms which were enforced upon themselves."

He refers to the unsuccessful endeavors of energetic medical reformers to have inserted into the medical act a clause punishing unqualified practitioners. The government would not accept any clause proposed to them, and the chief effect of the attempt was to bring into prominence the great difficulty of putting together any form of words which would fulfil the intended purpose, and which would not admit of being wrested into constructions improperly repressive of individual liberty. He ventured the opinion that the Medical Council could not punish for advertising, except the advertisement was so worded as to be of itself "infamous;" and questioned if they

---

[1] The same advance—for it is an advance—may be noted in our profession, producing the same result. Witness the increased productiveness of a pair of hands and one head as we have passed from carved plates to swaged plates; from hand-carved block teeth to the ready-made porcelain teeth of the dental depots produced commercially by the million; from the intricate and time consuming fabrication of metal and of porcelain dentures to those on cast or plastic bases. And, again, from contoured gold fillings, laboriously built up, to the plastics, to crowns and bridges, and to inlays. And, again, the lessened need of the dentist's care, the result of increased attention to preventative measures, and an increased appreciation of early and regular dental attention.

had the right to remove a name from the register for purely ethical offences. The law is primarily for the protection of the public, to enforce honesty and fair dealing, and has but slight regard for those rules or regulations the members of a calling or profession may see fit to adopt for their own pleasure or profit. If the law advances the material or social interests of the profession, it is a mere incident; it was not enacted for that purpose; indeed, in strict equity, could not be; nor can it justly be twisted or strained to effect that object. The profession must look to itself, and itself provide the ways and means of its own uplifting. While these sentiments may not be welcome, they probably forecast the final outcome of efforts on both sides of the sea to elevate by legal enactments the professional standard. This controversy between Mr. Horsley and Mr. Carter is well worth a careful reading, bearing in mind that while they are speaking particularly of the medical profession, all that is said is quite as applicable to the dental profession. The medical and the dental enactments are practically one, and both professions look to the same body to protect and to enforce their rights.

The controversy between these two gentlemen suggests much more than either have stated. While the dental law has undoubtedly had the desired effect of causing a much larger number than without it would have so done to appreciate the importance of proper preparation before entering professional ranks, and has done much, very much, to advance professional interests in every way; while, indeed, educationally, for the practitioner, the student, and the schools, it has done all that could reasonably have been expected from it, commercially, thus far, it has proved a failure. As read, it seems to be all that could be desired. When tested in court it has proved very defective. It is so worded (it is now said purposely so; for so jealously does the law-making bodies guard and protect the individual's rights and commercial interests, that had it been more stringently worded it would never have been enacted) that it is not only difficult to secure convictions, but a conviction usually carries with it nothing more than a small fine and the expense of a new sign or door-plate. It does not necessarily stop the business of the unqualified practitioner. This law has another serious defect in that it seems to allow companies and associations organized to carry on a dental business privileges and immunities denied to individuals. An earnest effort is now being made to correct this; but it is found to be a very difficult task to put together a form of words that will prove effective and yet be acceptable to the law-making powers.

The discussions upon this point, in the dental journals now in review, are well worth a careful reading.

In the July number, page 484, will be found a full report of the proceedings of the General Medical Council, the session beginning May 27, 1898, at which the Council recast the course of instruction and outlined the examinations recommended to the teaching and examining bodies for the qualification of L.D.S. A careful perusal of this will show how very different are the conditions encountered by a dental student in Great Britain and in the United States. While in Great Britain there are many dental and medical schools, they are simply teaching bodies. The examining and licensing bodies are separate and distinct. The dental student may obtain his education where he will, part at a medical school or hospital, and part at a dental school; part, if he so desires, in his preceptor's laboratory. It matters not, provided that the school is one approved by the Medical Council, and his preceptor is a qualified practitioner. He must, however, take his examinations from either the Royal College of Surgeons of England, the Royal College of Surgeons of Edinburgh, the Faculty of Physicians and Surgeon of Glascow, or the Royal College of Surgeons in Ireland; these are the only recognized examining bodies. So very different is the system of dental education in the two countries that it is no easy task for any one to compare them. Carefully going over this discussion, I am strongly impressed that the graded systematic course of the American dental college, where a continuous line of instruction is directed to the one object, preparing the student to enter the dental profession, which aims to impart all that is needed to be fully equipped to meet every phase of his life-work, where all the instruction he receives has this direct trend, is preferable to one such as is here recommended, "of shreds and patches," part dental and part medical; a great deal of which has to the dentist no practical usefulness, and which, taken as a whole, is, *apparently*, so unsystematic and disconnected that the chances are many things of vital importance will be overlooked, while others of great value are purposely omitted. As it has been planned and accepted by those who are presumed to have at heart the best interests of the profession, those who know better than we do the facilities at hand and the conditions their students are required to meet on entering actual practice, we may justly assume that it is the best course possible under the circumstances for a student who desires to practise in Great Britain, while at the same time holding firmly that the American Dental

College is infinitely better for one who intends to make the United States his home. I most earnestly commend a careful reading of this discussion and those which have led up to it to those interested in the international phase of dental education. It is far too important to be reviewed as briefly as this paper demands.

In passing, permit me to digress for a moment to suggest that this discussion, taken in connection with others in the same body which have from time to time preceded it, the many complaints from dentists, the retorts they have called forth from members of the medical profession which we find expressed in the journals, more forcible as the years roll round, have impressed me strongly that the dental profession in the United States have reason to be very thankful to those who first organized its educational system for striking out as they did on a new and independent line. In refusing to accept the meagre facilities grudgingly offered by the then medical colleges, and boldly and bravely assuming the task of organizing a school that should within itself teach all that a dentist is required to know; when they invented the distinctive degree, D.D.S.; when they cut loose from the medical profession with all its time-honored methods and traditions and proclaimed to the world their purpose to provide for their calling the means by which it should, unaided and alone, make for itself a position and a name among the professions of the world, they assumed a tremendous task; they made of it a grand success. This has made the dental profession in the United States not a branch, not a specialty, not a part of the great medical profession, but a profession of and in itself, separate and distinct, self-contained and self-supporting. We have not been trammelled in our educational advances as have been our brethren on the other side. Our educational pioneers were far too sturdy and self-reliant to patiently knock, year after year, subserviently seeking admission at the ponderous doors of the medical profession. They preferred to build a house of their own. We have thereby escaped the humiliation of being considered unwelcome guests; of being hampered in our pursuit of knowledge, and in the exercise of our skill and talent, by the jealousies of an elder brother. True, we have trenched upon the domain of surgery and medicine, as the Medical Council fear well-educated dentists will. We have taught both of these elder brethren lessons in methods and modes of procedure. Our mechanical skill, closely specialized observation, and delicate, well-trained finger craft have made their impress in unravelling histological mysteries; tracing the course of pathological changes; refining and modifying old and inventing new surgical appliances and processes. Graduates of American dental colleges have reversed the rule the General Medical Council would enforce if they could, in that they have been dentists first and surgeons after; and in the latter field, owing to their early training, have become recognized masters. Our educators are not controlled by commercially jealous masters having no sympathy with our profession, who enviously regard the golden ducats which a remote possibility may divert; who are not in touch with its marvellous progress, its possibilities, and its resources; but would cramp it within the narrow compass of "tooth-pulling and the like." Say what you will, deride it as you may, the American dental college has well earned its right to be. It has made a lasting impress on the world's onward and upward progress. That its graduates are so generally recognized as masters in their profession, and so many of them have risen to distinction, attest the thoroughness of its teaching and the completeness of its curriculum.

Considerable space is given to the proceedings of the Annual Meeting of the British Dental Association, at Bath, May 28–31, 1898, in the June number (page 343).[1] This is prefaced by an interesting illustrated account of this ancient Roman town, its hot springs, its baths, and its beautiful surroundings and historic associations. The Association has a membership of one thousand and nineteen, about one-fifth of those who are eligible, certainly a very creditable showing. The attendance is said to have been quite large, and the meeting, socially and educationally, satisfactory in every way. The finances of the Association are in excellent condition, it having, by the treasurer's report, some five thousand dollars on hand and invested. The Association takes a part in the financial burden of enforcing the dental laws, and this accumulated capital is a moral as well as a material help in carrying on this part of its work. In addition, separate and apart from its other work, the Association has taken care of its unfortunate members; indeed, with commendable broadness and liberality, it has now and again stepped over the line to extend a helping hand to those who have had no further claim than being followers of a common calling,—professional brothers in its widest sense. This is done by means of a "Benevolent Fund," maintained by yearly subscriptions, donations, etc., so well managed

---

[1] Journal of the British Dental Association, vol. xix., June, 1898, page 395.

and supported that its treasurer reported at this meeting an invested fund of over fifteen hundred pounds sterling. This has been accumulated from time to time over and beyond the amounts needed by those dependent upon it, and not only serves by the interest it earns to still further assist this charitable work, but gives to it a permanency, and stimulates professional pride and this noble and unselfish phase of professional loyalty.

During the time covered by the report, say about one year, some two thousand five hundred dollars were contributed to this fund. Of this, about two thousand dollars were distributed to disabled dentists, or the widows or children of dentists deceased. Nearly five hundred dollars of the year's receipts were carried to the investment account. The reports which have been from year to year published by the managers of this fund have been interesting reading. In a quiet way it has done a world of good. It has relieved dire distress, made comparatively comfortable the declining years of old practitioners and their wives, and assisted in the education of their dependent children. It is doing more than this. It is undoubtedly a factor in keeping up that *esprit de corps* which has brought into the national association nearly one-fifth of the legal practitioners in its bailiwick; this, when we consider that local dental associations or branches are scattered all over the land, is, indeed, an excellent record.

During the meeting Mr. Cunningham read the seventh report of a committee appointed by the Association to make a collective examination as to the condition of the teeth of school-children. It, as well as those which have preceded it, are well worth the attention of those interested. They are carefully written, and contain valuable information upon an interesting subject. They cannot, however, be usefully condensed without careful and close study.

The papers and demonstrations covered very well, indeed, the whole field of dental practice, and make a commendable showing of useful up-to-date work. Reluctantly passing the others for want of time, I ask attention to two that impress me as being especially suggestive. First, a paper by J. Smith Turner, M.R.C.S., L.D.S. (Eng.), entitled, "On Matters Connected with the British Dental Association," first answers the question, What is the use of registration? In so doing he defends those who arranged the details of the dental act, contending that they acted wisely in seeking to be attached to the Medical Council of Education, that body having already passed through the experimental stage with its unavoidable confusion and errors. By so doing the dental profession at once entered into a well-ordered and perfected system of registration. This he contended offset the initiatory errors of administration perpetrated by the Medical Council. It has been a long-standing grievance to the dental profession in Great Britain that while they have contributed largely to financially maintain the Medical Council, they have not been permitted representation in that body. It has required a long, persistent, determined struggle to acquire, by the appointment of Mr. Chas. S. Tomes, that which they have from the first considered their legal right,—to be represented by one of their own members in the body to whom they have committed the well-being of their calling.[1] To many the seeming indifference, the derogatory remarks, and the dilatory manner associated with dental business in the Medical Council has raised the question whether association with that body has not been a determent; whether it would not have been better to have "gone it alone." It is to these Mr. Turner first addresses his remarks. He next turns to the student who has just received his diploma, and urges him not to rest satisfied with the honors it confers, but to at once make his title legally and honestly complete by the act of registration. He next urges the importance of being enrolled as a member of the national body, an association that, notwithstanding its shortcomings, is the embodiment of the wisdom of the dental profession, and has on its roll of members all that is best and all that is noteworthy in our calling on both sides of the Tweed and in the sister island. He then refers to the advantages offered by the national association, socially, professionally, and educationally; notes the inconvenience to those residing remotely from the place of meeting, no matter where the place of meeting may be, an effort to attend its meetings involves; and closes with an earnest plea that, in addition to membership in the central body and attendance on its yearly gatherings whenever possible, as a supplement thereto, membership in and active co-operation with some one of the local branches. He suggests that the local branches should be so located, and their area so restricted, as to, as much as possible, make their meetings readily accessible to all their members. Useful as they undoubtedly are, however, they are necessarily local, he contends, and have the tone and color of a local meeting. They lack that subtle influence, the broad and liberal views, which so greatly modify local prejudices,

---

[1] Dental Record, vol. xviii., April, 1898, page 154.

and the cosmopolitan character which gives to the annual meetings of the National Association so great an impetus to mental activity and expansion.

Mr. F. J. Bennett, M.R.C.S., L.D.S. (Eng.), in a paper entitled "The Ending and Mending of Caries," suggests that the old maxim, which says, "What can't be ended must be mended," represents the point of view from which a large majority of dentists regard the subject of dental caries. They cannot end the advent of caries, therefore they devote all their energies to mending its destructive effects. He further suggests that the success in mending has tended to lessen the desire to end, or, rather, in the sense in which he uses this word, to prevent that which makes mending necessary. Had there been less resource in the mending; had the case admitted of no half-measures; had the dangers of caries been more deadly, more widespread attention and an unremitting effort to the discovery of a prevention and ending of caries would have been made. He reviewed the efforts made to discover the cause of caries which have led up to the present accepted theories, and, reasoning from the success attending preventative measures in lessening other germ-caused diseases, urged that there was in this sufficient encouragement to stimulate effort to find some practical means of lessening if not ending the necessity of mending dental caries.[1]

The social features of the meeting appeared to have been especially pleasant. Owing to the serious illness of the president, Dr. Stack, Sir Edwin Saunders was called upon to take his place. The approaching fiftieth anniversary of his wedding was pleasantly alluded to, and later on was fittingly celebrated.[2] Next to the late Sir John Tomes, Sir Edwin Saunders has been foremost in making dentistry in his native land the respected calling it is to-day. His presence at any dental gathering will ever be an inspiration.

The well-known *British Journal of Dental Science* completed with the close of the year its forty-first volume. It lacks the stateliness of the official journal; its contents are more varied, and largely drawn from other journals. They are, however, in the main, judiciously selected and properly credited. Its original matter is rather largely composed of papers written by students and read before student societies. In the July number, page 644, this is editorially noted as having been complained of by subscribers, and is

[1] Journal of the British Dental Association, vol. xix., August, 1898, page 601.

[2] Ibid., vol. xix., 1898, page 789.

there defended, the writer saying, "We must not forget that we were all students once, and must also remember that it is a great incentive to a student to take the trouble to compile a paper when he feels that what he has written will appear in a journal read by those among whom he hopes shortly to rank." There is doubtless truth in this, and possibly the kindly reception of these maiden efforts and the encouragement thus given may be followed in time by more serious and more matured contributions that otherwise the profession would miss. The youngster who to-day quotes so largely from his text-books, and who is encouraged by seeing his paper in print to continue his contributions to his profession's literature, may shortly with equal zeal pry into nature's secrets or write text-books himself. I am impressed, however, that the college journals so common on this side, dental journals published by students for students, more fully "foots the bill." The publication of such papers in a journal intended for practitioners, by crowding out more valuable matter, makes it less valuable to those for whom it is intended, who have no interest in these juvenile essays. It also leads more experienced writers to avoid it.

Its editorials are usually well written, and are upon topics of general interest. One in the number for May 15, page 458, upon "Reciprocity," commends the work of our National Association of Dental Faculties in levelling up the educational standard of dentistry in the United States. It compares their work with that of the General Medical Council, which is doing the same for the profession in Great Britain. The writer has, of course, his little fling at American dental colleges; nevertheless, he looks upon these two bodies as preparing the way, perhaps it may be in the far distant future, for international educational reciprocity. It probably may not, he suggests, come all at once. It may be that in this interchange of diplomas they may be accepted first in part only, the holder being required to make up by added study that portion of the curriculum his diploma does not cover. He makes no note of the commercial features which have in this so large a part, and are becoming more and more controlling factors in all forms of international reciprocity.

The *Dental Record*, a monthly journal published by the Dental Manufacturing Company, in the information it furnishes, caters more closely to the dentist's practical needs than either of those I have noticed. On page 55, W. H. Gilmore, L.D.S., treats of the "Debatable Points in Anæsthesia." The subject of anæsthesia has with them occupied a large share of professional thought. Notwithstanding frequent fatalities, chloroform seems to be largely used on the other side, and was responsible for nearly one hundred deaths during 1898 in Great Britain alone. When we remember that it is in most cases administered by a medical practitioner, the dentist not being educated, it is supposed, sufficiently far in medical matters to be trusted with the administration of anæsthetics, these repeated records of death under anæsthetics in the dental chair are astounding. It has been questioned whether, indeed, the caution is not really responsible for this alarming fatality. Withholding from the dental student the information and the *practice* needed to make him fully conversant with the phenomena associated with anæsthesia for dental purposes and in the dental chair, and assigning this delicate operation to one who, however skilful in its use at the surgical clinic or the parturient couch, is here entirely out of his element, seems to be a serious mistake, too often a fatal one to the unfortunate patient. When we remember that anæsthesia was introduced by dentists, its lines of safety and of danger mapped out by dentists, the most approved instruments for its use invented by dentists, it seems very strange to raise the question whether or not a dentist should be allowed to administer it. And yet this question is, on the other side, not only frequently discussed, but is generally decided that his medical education is too limited if his qualification is dental only; and yet they charge that a graduate of an American dental college whose right to do this has never on this side been challenged, and who has for half a century fully proved his competency to so do with safety and success, is, compared with their graduates, an imperfectly educated man! The debatable points noted by this writer are, whether nitrous oxide has been more a blessing or a curse; whether, when a medical man is called in to administer ether or chloroform, it should be the patient's ordinary medical attendant or an expert anæsthetist selected by the dentist; lastly, who shall choose the anæsthetic? Regarding this last, he holds that the dentist should be allowed to ask for any anæsthetic he prefers. If, however, the medical man should decide that the patient's physical condition contraindicates the anæsthetic chosen, the dentist must then give way, placing the whole responsibility upon the anæsthetist. The latter seem to have become specialists, having a society of their own. Complaint has been made that medical men fail to appreciate the importance of those precautions and that care usually surrounding the use of anæsthetics in surgical cases when called upon to use an anæsthetic for tooth extraction. In the presence of the surgeon the anæsthetist is the assistant; in the presence of the dentist he is too apt to feel that he is the *master*, and is there to "boss the job." I note a variety of devices to prolong the effect of nitrous oxide without increasing the danger of its use. Some seek to accomplish this by continuing in various ways its administration through the nose or mouth during the operation; others by combining it with ether, or by using a little ether immediately after the gas. Those interested in this matter will find several well-written articles describing these methods in last year's *Dental Record.*

On page 251 a patented artificial or dummy patient for students to practise on is illustrated, and in a later number appears a dispute as to who first introduced the device. Before settling that point it will be well for the contestants to confer with our friend, Professor J. Foster Flagg, who for many years used the same ingenious device in his lectures and clinics.

Taking it all in all, as a practical journal, the *Dental Record* deserves first place among the English dental journals, and has, during the year under review, kept its readers as well informed as one journal can of all matters of current interest concerning the practice of dentistry and the dental profession.

The *Dentist*, published monthly by Hampton & Co., 13 Cursitor Street, London, England, a new venture, made its appearance January, 1898. It claims to be an independent journal devoted to the interests of the dental profession.

I note a new feature, and perhaps a desirable one. Each number begins with a few pages entitled "Notes of the Month." These are a series of disconnected paragraphs noting recent events, professional and otherwise, of general or local interest, or directing attention to new ideas or devices, or to articles or special points brought out by contributors in the current number. They are not abstracts from other journals, but brief, original, newsy notes. Judiciously edited, I am impressed, it may be made a desirable feature,—one not uncommon in unprofessional periodical literature, and usually appreciated.

Chloroform, as an anæsthetic, is discussed at some length in the earlier numbers, especially in reference to Dr. Snow's method of administration, and the deductions his long experience in its use and close observation of its peculiar effects led him to formulate. In one of the monthly notes (April, page 118) he is styled "The Father of Anæsthesia"(?), and it is there suggested that a monument to him should be erected in the city of York, where he was born. Another note on the same page reads as follows: "The most emphatic testimony yet borne to the value of Snow's life-work is the fact that no one has disproved the truth of his conclusion that twelve minims of chloroform, slow-progressively administered, can have no other effect in an adult than loss of sensation without loss of consciousness. Eighteen minims, loss of sensation, consciousness, and motion,—all that is required in any ordinary surgical operation. Twenty-four minims uniformly diffused in the blood produces thorough relaxation of the muscles, only required for the reduction of long-standing dislocations; and further than this, Snow's fourth degree, chloroform should never be pushed."

The writer contends that had this rule been observed from the first not a single accident would have occurred from its over-administration, to which alone, he says, death from chloroform is due. On page 54 is an article describing an apparatus, made by Mr. Krohne, designed to carry out the Snow method, and on page 120 this is further explained and illustrated. The object sought is to know visually how much and how rapidly the anæsthetic is being given and the relation it bears to the atmospheric air given with it.

While the first volume of this new journal contains much of value, there is much room for improvement. It can hardly be considered, in any respect, a better professional journal than its competitors. It began its second volume as a weekly.

Glancing over the field the two most important events of the year have undoubtedly been the appointment for five years of Mr. Chas. S. Tomes as a dental representative upon the General Medical Council, fortunately, owing to the death of Sir Richard Quain having made an opening, in time to take part in the second important event, settling the long unsettled dental curriculum in so far as the General Medical Council's authority can so do. Its recommendations are only advisory; they will, however, no doubt, be as closely followed as circumstances permit.

In conclusion. Quite a large space in all the journals is given to reports of actions at law to enforce the various phases of the Dental Act. While this may be wise to a certain extent, they do not, so fully and so frequently reported, make interesting reading. When in addition the private misfortunes of dentists, which have no bearing whatever on professional matters, bankrupt proceedings, infractions of the moral law, divorce scandals, business disagreements or misunderstandings, or other legal matters in which a dentist, through fault of himself or of others, is brought, willingly or unwillingly, into court, are thus repeated, they seem very much out of place in a dental journal. I am very sure it would not be tolerated on this side. It is not my purpose, however, to criticise; but rather to prompt a more general reading of these journals, and thus promote a more generous interchange and understanding of international dental thought.

